# Are adverse childhood experiences associated with trajectories of healthy aging? Evidence from China

**DOI:** 10.1016/j.ssmph.2023.101501

**Published:** 2023-08-26

**Authors:** Ju Sun, Jingru Wang, Haomiao Li

**Affiliations:** School of Political Science and Public Administration, Wuhan University, Wuhan, China

**Keywords:** Adverse childhood experiences, Healthy aging, Trajectory

## Abstract

Adverse childhood experiences (ACEs) have life-course impact and may be associated with healthy aging. This study aimed to explore the association between ACEs and healthy aging trajectories among middle- and older-aged adults in China. The data were obtained from the China Health and Retirement Longitudinal Study from 2011 to 2018. A total of 8906 respondents were enrolled in the analysis. Through latent growth mixture model, five categories of healthy aging trajectories which defined as ‘stable type with high starting point’, ‘stable type at the middle level’, ‘stable type with low starting point’, ‘rapid descending type with high starting point’, and ‘stable descending type with medium starting point’ were identified. Based on multinomial logistic regression, ACEs were significantly associated with healthy aging trajectories (stable type at the middle level/stable type with high starting point: relative risk reduction [RRR] = 1.19; P < 0.01; 95% CI = 1.16–1.23; stable type with low starting point/stable type with high starting point: RRR = 1.35; P < 0.01; 95%CI = 1.21–1.51; rapid descending type with high starting point/stable type with high starting point: RRR = 1.09; P < 0.1; 95% CI = 0.99–1.19; and stable descending type with medium starting point/stable type with high starting point: RRR = 1.30; P < 0.01; 95% CI = 1.23–1.38). When treating ACEs as a categorical variable, the healthy aging trajectory of the rapid descending type with a high starting point was not affected by any ACE groups. Further analysis of the relationship between each ACE and the trajectories of healthy aging reveals that parental disability, household mental illness, domestic violence, physical abuse, unsafe neighbourhood and bullying had significant impacts on almost every developmental trajectory of unhealthy aging. The government should promote the realisation of healthy aging from the perspective of the entire population and life cycle, intervene early in life, avoid the occurrence of ACEs as much as possible, and minimize the harm done by ACEs as much as possible.

## Highlights

• Adverse childhood experiences (ACEs) may have lifelong impact on individual’s health.
• The association between ACEs trajectory of health aging have not been well investigated.
• The number of ACEs was significantly related to unhealthy aging trajectories.
• The rapid descending type of ACEs with high starting point was not affected by any ACE groups.

## 1 Introduction

Global population aging has become a major trend in the 21st century development. With the continuous acceleration of aging, the average life expectancy is prolonged, and health conditions are improving; however, the growth rate of life expectancy is slightly faster than that of Heath-Adjusted Life Expectancy (HALE) (WHO, 2022). Therefore, promoting healthy aging has become the most important national policy in many countries. In 2015, the World Health Organization (WHO) released the Global Report on Aging and Health, which defined healthy aging as “the process of developing and maintaining the functional ability that enables well-being in older age” (WHO, 2015). In May 2020, the United Nations Decade of Action for Healthy Aging called for a decade of coordinated and healthy aging actions worldwide to address population aging (WHO, 2020).

According to data from the National Health Commission of the People's Republic of China (PRC), approximately 190 million older population in China suffered from chronic diseases, with around 40 million experiencing disability or semi-disability, and approximately 15 million diagnosed with dementia. The problem of unhealthy longevity has become increasingly prominent in China as it has the largest older population (Lancet T, 2016). China has paid great attention to healthy aging strategies and has incorporated them into the overall strategic layout of the country. In March 2017, the 13th Five-Year Plan for Healthy Aging gave a definition of ‘healthy aging’ in China as follows: ‘from the perspective of the whole life process, from the early stage of life, we should comprehensively and systematically intervene all the factors that affect health, and create social support and living environment conducive to the health of the older population, so as to prolong the healthy life expectancy, safeguard the health function of the older population and improve their health level’.

Both the international and Chinese concepts of healthy aging emphasise the common points of promoting healthy aging from the perspective of the whole population and whole life cycle and induce not a static state but a dynamic and diversified process. Accordingly, healthy aging in individuals is influenced by their internal abilities and environment. With changes in an individual's internal abilities and the external environment, the level of healthy aging also presents different trends (Stephens et al., 2019; Li et al., 2021; Liu et al., 2021). Based on the heterogeneity of older population and dynamic changes in healthy aging levels, the trajectory of healthy aging may present heterogeneity among groups. Therefore, monitoring and evaluating the trajectory of change and its influencing factors are of great significance for the formulation and implementation of relevant policies and intervention measures.

Based on the theory of life course, childhood is an important and necessary stage for everyone to grow, as well as a particularly sensitive period that is most easily influenced by the external environment. Happiness and misfortune during childhood may play an extremely important role in shaping the health status of individuals throughout their lives. A substantial number of studies have explored the associations between adverse childhood experiences (ACEs) and deleterious health consequences, including risk of frailty, accelerated development of biological aging, chronic diseases and multimorbidity, depression, risk of cognitive dysfunction, and so on (Baranyi et al., 2019; Colich et al., 2020; Gold et al., 2021; Lin et al., 2021; Rocca et al., 2017; Xie et al., 2022; Yao, 2021; Zhang & Zhang, 2022). Some studies have further explored the possible mechanisms between ACEs and healthy outcomes. Some scholars have found that the relationship between ACEs and healthy outcomes is mainly through the modulation mechanism of biological aging such as telomere length, mitochondrial function, pubertal timing, alterations in fetal and infant development, conveyance of childhood chronic illness into adulthood, stress reactivity and inflammation, etc (Wise, 2016; Yang et al., 2022). Others have found that unhealthy lifestyles (body mass index, smoking status, alcohol consumption, physical activity and diet) played a partial mediating role in that association (Yang et al., 2022; Morton et al., 2022). In addition, some possible mediators have also been widely concerned by scholars, such as personal education level and personal income, childhood health status, childhood economic status, and inadequate access to effective interventions in childhood, including childhood health investment and childhood socioeconomic status (Morton, 2022; Tao et al., 2021). Previous studies indicated that ACEs may be associated with the trajectory of healthy aging. Nevertheless, existing studies have mainly focused on a certain aspect of health and lack a comprehensive index to measure healthy aging. Moreover, most current studies were cross-sectional, which does not reflect the dynamic changes in health among older populations. However, the association between ACEs and the trajectory of healthy aging has not been thoroughly investigated (de la Fuente et al., 2018; Hu, 2021; Jiang et al., 2022; Li et al., 2022; Morton, 2022; Wise, 2016).

In this study, we investigated whether there was an association between the trajectories of healthy aging and ACEs. We focused on the influence of ACEs on the trajectories of healthy aging from a dynamic life-cycle perspective based on longitudinal data, which not only enriched the existing research on healthy aging, but also provided evidence and a decision-making reference for realising the strategy of actively coping with population aging and healthy aging.

## 2 Methods

### 2.1 Data source and sample selection

The data used for this study were obtained from the China Health and Retirement Longitudinal Study (CHARLS), which was conducted by the National School of Development of Peking University in 2011, 2013, 2015, and 2018 using a multistage stratified probability-scale proportional sampling method to select interviewees among Chinese residents aged ≥45 years. Information on ACEs was collected from the 2014 Life History Survey. Details of all data can be found on the official website (http://charls.pku. edu.cn/). The Biomedical Ethics Review Board of Peking University approved CHARLS (approval number: IRB00001052–11015). Data analysis was performed from 1 to 31 January 2023. After excluding those with incomplete or inapplicable data, 8906 people were included in the analysis (Supplementary Fig. S1).

### 2.2 Variables

#### 2.2.1 Measurement of healthy aging

Based on the WHO framework and previous studies (Beard et al., 2016; Li et al., 2022; Wu et al., 2020), we identified 41 items (Supplementary Table S1) related to activities of daily living (ADL), Instrumental activities of daily living (IADL), psychological symptoms, cognitive function, vitality, and sensory and mobility problems, which may indicate the underlying concept of healthy aging. The selected items were dichotomised into binary variables (0 = presence of difficulty and 1 = absence of difficulty).

The item response theory (IRT) was used to calculate scores for healthy aging. Compared to the traditional approach of calculating a total score, IRT models can overcome some limitations of classical measurement theory, take into account variations in response patterns, and generate corresponding latent trait scores to reflect such variations, which convert the values of potential characteristic variables and numerical values of item difficulty parameters that cannot be directly observed by different individuals into the same ability scale for comparison. Two-parameter logistic IRT was used to incorporate 41 items and estimate the latent trait scores for respondents. The estimated latent trait score followed a normal distribution at a range of 0–1. The parameters estimated from baseline data were applied to the follow-up waves under the assumption of measurement invariance over time. To improve the interpretation of results, the latent trait scores were rescaled to a range between 0 and 100, with higher scores indicating healthier aging.

To check the reliability of the healthy aging scores, we further calculated empirical reliability using a Cronbach’s alpha coefficient of 0.84, and marginal reliability using KMO and Bartlett's test. KMO takes values between 0 and 1, and a value of 0.92 near 1 indicates a good fit for factor analysis. The approximate chi-square value of Bartlett’s test was 195257.72 and P < 0.001, which indicated that the survey data had a high correlation and were suitable for factor analysis.

#### 2.2.2 Adverse childhood experiences

The childhood period typically refers to the stage spanning from birth to adolescence. Consistent with previous researches (Lin et al., 2021; Wang, 2021), utilizing the questionnaire design based on CHARLS data, childhood is defined as the period from birth until the age of 17, inclusive. Information on childhood adversities before 17 years were collected through face-to-face interviews in the 2014 life history survey. Twelve ACEs were selected for this study: physical abuse, emotional neglect, household substance abuse, household mental illness, domestic violence, incarcerated household members, parental separation or divorce, unsafe neighbourhoods, bullying, parental death, sibling death, and parental disability. The detailed questionnaire items and definitions for each ACE indicator are provided in Supplementary Table S2. Responses to each item were dichotomised and exposures to ACEs reported by each participant was summed to generate a cumulative total ACE scores for each participant, ranging from 0 to 12. We further categorised the participants into 5 groups based on their cumulative ACE scores: 0, 1, 2, 3, and ≥4 (Lin et al., 2021).

#### 2.2.3 Covariates

To minimize bias in estimating the association between ACEs and trajectories of healthy aging, directed acyclic graphs were applied to select the appropriate adjustment set of covariates using DAGitty v3.0. The final minimally sufficient adjustment set consisted of age, sex, marital status, educational level, rural or urban residence, current work status, annual per capita household expenditure level, childhood economic hardship, childhood health status, smoking status, drinking status, social participation, social security (medical insurance and pension), and weekly contact with children, as in the model at the individual and family levels obtained from data in 2011 (Fig. S2). Further details on these variables are provided in Table 1.

**Table 1:** Sample description (2011).

Variables	Mean ± SD/N(%)
**ACEs scores**	2.80 ± 1.89
**ACEs group**	
0	787(8.84%)
1	1656(18.59%)
2	1953(21.93%)
3	1701(19.10%)
≥4	2809(31.54%)
**Age**	57.71 ± 8.18
**Gender**	
male	4143(46.52%)
female	4763(53.48%)
**Marital status**	
Married with spouse present	7658(85.99%)
others	1248(14.01%)
**Educational level**	
Less than lower secondary education	7972(89.51%)
Upper secondary & vocational training	837(9.40%)
Tertiary education	97(1.09%)
**Area of residence**	
Rural	5846(65.64%)
Urban	3060(34.36%)
**Current work status**	
Not working	2735(30.71%)
Working	6171(69.29%)
**Childhood economic hardship**	
Yes	3589(40.30%)
No	5317(59.70%)
**Poor Childhood health status**	
Yes	1135(12.74%)
No	7771(87.26%)
**Covered by Public Health Insurance Program**	
None	545(6.12%)
Yes	8361(93.88%)
**Received Any Public Pension**	
None	7378(82.84%)
yes	1528(17.16%)
**Take Household per capita consumption's log**	7.28 ± 3.05
**Smoking status**	
Never	5469(61.41%)
Quit now	727(8.16%)
Still	2710(30.43%)
**Drinking status**	
Never	5243(58.87%)
Quit now	913(10.25%)
Still	2750(30.88%)
**Participated in social activities**	
None	4752(53.36%)
yes	4154(46.64%)
**Weekly contact with children**	
None	833(9.35%)
yes	8073(90.65%)

*Note:* ACEs, adverse childhood experiences.

### 2.3 Statistical analysis

In the description analysis of the respondents’ baseline characteristics, “percentage” was used for the description of binary or categorical variables, and mean ± standard deviation (SD) was used for the description of continuous variables with normal and abnormal distribution.

We applied the latent growth mixture model (LGMM) to classify the development trajectory groups of the healthy aging scores of the respondents across the four survey waves and test the predictors of membership in these classes. LGMM was first proposed by Muthen et al. (1999), and it is a longitudinal data analysis method to identify the changing trend of longitudinal data. When the development trends of the observations are inconsistent, LGMM can make up for the deficiency of traditional growth model in discussing group heterogeneity. LGMM assumes that there are multiple potential growth trajectories in the observations, with each potential trajectory representing a subcategory, and the growth patterns of different subcategories are different, which means that the research object is allowed to be heterogeneous. A series of parameters of dividing into 2–6 classes, including the Bayesian information criterion (BIC), the Akaike information criterion (AIC), entropy values, and the Lo-Mendell-Rubin likelihood ratio test (LRT), were used to select optimal classification.

We further applied a multinomial logistic regression model (MLRM) to explore the relationship between the developmental trajectories of healthy aging and ACEs with covariates controlled. The effects of the scores and categories of ACEs were analysed. Further analysis of the relationship between each ACE and the trajectories of healthy aging were conducted to explore the mechanisms and provide more accurate suggestions. It should be noted that, as shown in Supplementary Fig. S1, more than 60% participants were excluded from this study, which may raise concerns about the representativeness of the sample and the generalizability of the results. In term of this, the study incorporated sampling weights to perform weighted analysis aiming to obtain more accurate results and minimize estimation errors.

To verify the reliability and robustness of our research conclusion, we conducted several sensitivity analyses. First, based on the 2011 data, additional control variables, namely self-rated health and chronic disease were included in the model. Second, we added additional self-rated health and chronic disease to main model using 2018 data without incorporating sampling weights to repeat the above analysis. Third, a random-effects model was used to investigate the influence of ACEs on healthy aging scores. Fourth, considering that the impact of ACEs on healthy aging trajectories may vary across individuals, we explored the heterogeneity of the relationship between ACEs and the trajectories of healthy aging among urban/rural residence, gender, and across middle-aged (aged 45 to 59) and older population (aged 60 and above) groups. A two-sided P-value <0.05 was considered statistically significant. Except for the LGMM model analysed by Mplus Editor 8 (http://www.statmodel.com/), most remaining statistical analyses were conducted using Stata version 15.1 (StataCorp LLC).

## 3 Results

Table 1 shows the descriptive statistics of variables used at baseline (2011). Of the 8906 participants included, the baseline population had a mean healthy aging score of 66.62. Only 8.84% of respondents had never experienced an ACE, and each respondent had suffered more than two ACEs on average. There were 4763 (53.48%) females and 4143 (46.52%) male respondents. The mean (SD) age was 57.71 (8.18) years. Furthermore, 89.51% of the respondents had only junior school education or below, mainly (65.64%) living in rural areas, and more than two-thirds (69.29%) were still working; 12.74% of the respondents had poor childhood health status (level), 40.3% had a poor childhood financial situation. Moreover, 93.88% of the respondents covered by the Public Health Insurance Program, and 82.84% had not received any public pension. Most interviewees never smoked or drank alcohol, but more than half did not participate in social activities. A high proportion (90.65%) of respondents were able to keep in touch with their children every week.

**Fig. 1:**
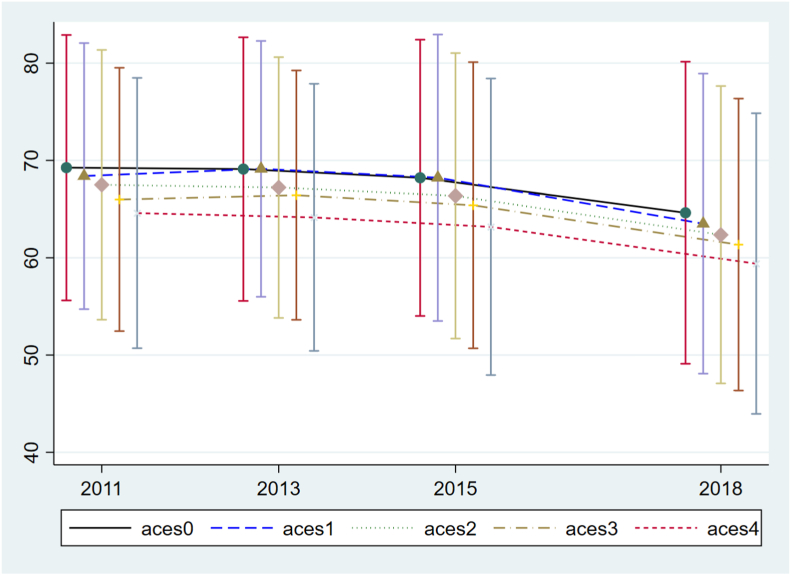
Healthy aging score among different ACE groups.

Fig. 1 shows that the trend of average healthy aging score varies with different ACE groups. The average level of healthy aging score in the five ACEs groups all showed a gradually decreased trend with time, and the trends were almost parallel. Moreover, the starting point (average healthy aging scores in the base period) of the group with the most ACEs is the lowest.

Table 2 presents the results of LGMM. Using a latent growth mixture model, we compared 2–6 classes of unconditional models for the development trajectories of healthy aging scores after adjusting for age and sex and examined the BIC, AIC, entropy values, and LRT. We sought a model with lower values for the criterion indices, higher entropy values, and LRT P value. The results suggested that the five-class solution was the best.

**Table 2:** Fit indices for latent growth mixture models.

	AIC	BIC	Entropy	Lo-Mendell-Rubin test *P* value
2 classes	272329.66	272450.26	0.52	***
3 classes	272102.48	272251.46	0.59	***
4 classes	271900.29	272077.65	0.65	***
5 classes	271848.65	272054.39	0.69	***
6 classes	271800.47	272034.59	0.63	0.05*

*Note:* AIC, Akaike information criterion; BIC, Bayesian information criterion.

*, **, *** statistically significant at the 10%, 5% and 1% levels, respectively.

**Fig. 2:**
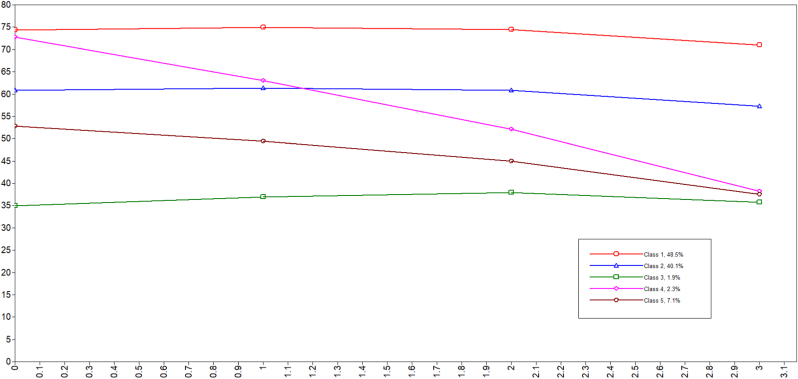
Trajectory of healthy aging.

As shown in Fig. 2, five groups of healthy aging trajectories were observed, and the trajectories of healthy aging of the respondents were not the same. Thereafter, we defined the five classes as ‘stable type with high starting point’, ‘stable type at the middle level’, ‘stable type with low starting point’, ‘rapid descending type with high starting point’, and ‘stable descending type with medium starting point’ to represent the trajectories of healthy aging. Class 4 (rapidly declining type with high starting point) exhibited the strongest decreasing trend. The trajectories of classes 2 and 3 were approximately parallel to class 1 but had lower starting points. Supplementary Table S3 further demonstrates the characteristics of five types of healthy aging trajectories. The group in the stable type with high starting point trajectory did not exhibit the lowest average age, but exhibit the lowest prevalence of chronic diseases among the five trajectory groups. Moreover, they suffered the fewest average scores of ACEs, and had the highest proportion of urban residents. In addition, the group with the lowest average age was found in the trajectory of ‘stable type at the middle level’.

Considering class 1 (the best group: stable type with a high starting point) as a reference, Table 3 shows the influence of the ACE scores on the healthy aging trajectories incorporating sampling weights. The relationship between ACEs and healthy aging trajectories remains significant, regardless of the inclusion of covariates. Respondents with greater scores of ACEs had a significantly higher likelihood of being having a poor healthy aging trajectory (stable type at the middle level/stable type with high starting point: relative risk reduction [RRR] = 1.19; P < 0.01; 95% CI = 1.16–1.23; stable type with low starting point/stable type with high starting point: RRR = 1.35; P < 0.01; 95%CI = 1.21–1.51; rapid descending type with high starting point/stable type with high starting point: RRR = 1.09; P < 0.1; 95% CI = 0.99–1.19; and stable descending type with medium starting point/stable type with high starting point: RRR = 1.30; P < 0.01; 95% CI = 1.23–1.38). Moreover, there is a complex relationship between different groups of ACEs and healthy aging trajectories. The healthy aging trajectory of the stable type at the middle level was affected by two or more ACEs; the healthy aging trajectory of the stable type with a low starting point was affected by four or more ACEs, and the healthy aging trajectory of the stable descending type with a medium starting point was affected by three or more ACEs. However, the healthy aging trajectory of the rapidly descending type with a high starting point was not affected in any of the ACE groups (details presented in Table 3).

**Table 3:** The association between ACEs and trajectory of healthy aging based on multinomial logistic regression.

Variable	Stable type at the middle level				Stable type with low starting point				Rapid descending type with high starting point				Stable descending type with medium starting point			
	RRR (95% CI)	RRR (95% CI)	RRR (95% CI)	RRR (95% CI)	RRR (95% CI)	RRR (95% CI)	RRR (95% CI)	RRR (95% CI)	RRR (95% CI)	RRR (95% CI)	RRR (95% CI)	RRR (95% CI)	RRR (95% CI)	RRR (95% CI)	RRR (95% CI)	RRR (95% CI)
**ACEs scores**	–	1.11*** (1.08, 1.14)	–	1.19*** (1.16, 1.23)	–	1.30*** (1.17, 1.44)	–	1.35*** (1.21, 1.51)	–	1.07 (0.98, 1.16)	–	1.09* (0.99, 1.19)	–	1.22*** (1.16, 1.28)	–	1.30*** (1.23, 1.38)
**Aces(Categorical Variable, Ref: aces=0)**																
**1**	0.95 (0.78, 1.15)	–	1.03 (0.82, 1.28	–	1.43 (0.56, 3.63)	–	1.65 (0.65, 4.17)	–	1.05 (0.50, 2.20)	–	1.11 (0.53, 2.33)	–	1.17 (0.75, 1.84)	–	1.27 (0.79, 2.04)	–
**2**	1.09 (0.90, 1.33)	–	1.37*** (1.11, 1.71	–	1.67 (0.67, 4.17)	–	2.02 (0.80, 5.07)	–	1.19 (0.58, 2.45)	–	1.33 (0.65, 2.72)	–	1.12 (0.72, 1.74)	–	1.41 (0.89, 2.24)	–
**3**	1.35*** (1.11, 1.65)	–	1.88*** (1.51, 2.35	–	0.91 (0.33, 2.53)	–	1.17 (0.41, 3.29)	–	1.65 (0.78, 3.49)	–	1.92* (0.91, 4.03)	–	1.70** (1.10, 2.64)	–	2.38*** (1.50, 3.79)	–
**≥4**	1.48*** (1.23, 1.78)	–	2.17*** (1.74, 2.70	–	2.90** (1.22, 6.87)	–	3.59*** (1.49, 8.65)	–	1.29 (0.65, 2.56)	–	1.46 (0.72, 2.93)	–	2.37*** (1.57, 3.56)	–	3.39*** (2.17, 5.23)	–
**Covariates**	uncontrolled	uncontrolled	controlled	controlled	uncontrolled	uncontrolled	controlled	controlled	uncontrolled	uncontrolled	controlled	controlled	uncontrolled	uncontrolled	controlled	controlled

Note: Reference: stable type with high starting point.

RRR Relative Risk Ratio. *, **, *** statistically significant at the 10%, 5% and 1% levels, respectively.

Sampling weights were taken into analysis.

Supplementary Table S4 shows the relationship between each type of ACE and the trajectories of healthy aging. Overall, the majority of ACEs, such as parental disability, household mental illness, domestic violence, physical abuse, unsafe neighbourhood and bullying had a significant impact on almost every developmental trajectory of unhealthy aging.

The results of the sensitivity analyses are presented in Supplementary Table S5. When adding control variables such as chronic diseases and self-rated health in the model and incorporating sampling weights, the research results were consistent with the main model (Table 3). Controlling for all covariates including chronic diseases and self-rated health in 2018, and without considering sampling weights, the results of the MLRM were also consistent with those of the main analysis, validating our conclusions. Additionally, we applied a random-effects model to assess the impact of ACEs on healthy aging scores, accounting for time-varying covariates. The results also showed that respondents who suffered more ACEs incidents were more likely to have lower healthy aging score (β = −0.79; P < 0.01).

Table 4 shows the results of heterogeneity analysis of gender, age and urban-rural residence, respectively. In terms of age groups, using the stable type with high starting point trajectory as the reference similarly, ACEs were significantly associated with the trajectory of healthy aging for the stable type at the middle level among the middle-aged population (aged 45 to 59), and had a significant effect on the trajectory of healthy aging for stable descending type with medium starting point among older adults (aged 60 and over). Rural residents who had experienced two or more ACEs exhibited significant differences in their trajectories of healthy aging, while urban residents who had experienced three or more ACEs exhibited significant differences. Furthermore, when examining differences by gender, females who had experienced two or more ACEs exhibited significant differences in their trajectories of healthy aging, while males who had experienced three or more ACEs exhibited significant differences among the trajectories of healthy aging.

**Table 4:** Heterogeneity analysis of gender, age and rural/urban residence.

	Male	Female	middle-aged individuals	older population	urban	rural
**ACE scores**						
Stable type at the middle level	1.19*** (1.14, 1.24)	1.20*** (1.14, 1.25)	1.14*** (1.10, 1.18)	1.18*** (1.12, 1.24)	1.12*** (1.06, 1.19)	1.23*** (1.18, 1.28)
Stable type with low starting point	1.35*** (1.12, 1.61)	1.36*** (1.18, 1.57)	1.38*** (1.21, 1.58)	1.23** (1.02, 1.48)	1.49*** (1.25, 1.78)	1.30*** (1.12, 1.50)
Rapid descending type with high starting point	1.04 (0.92, 1.17)	1.15* (0.99, 1.32)	1.06 (0.94, 1.19)	1.09 (0.95, 1.25)	1.15* (0.98, 1.36)	1.07 (0.96, 1.20)
Stable descending type with medium starting point	1.32*** (1.67, 1.50)	1.31*** (1.22, 1.40)	1.23*** (1.14, 1.32)	1.27*** (1.18, 1.38)	1.41*** (1.24, 1.61)	1.30*** (1.22, 1.38)
**Aces(Categorical Variable, Ref: aces=0)**						
***1***						
Stable type at the middle level	1.14 (0.74, 1.77)	0.94 (0.73, 1.22)	1.12 (0.85, 1.46)	0.98 (0.69, 1.37)	1.01 (0.69, 1.48)	1.03 (0.78, 1.35)
Stable type with low starting point	0.93 (0.23, 3.73)	2.09 (0.58, 7.55)	1.96 (0.54, 7.20)	1.40 (0.38, 5.16)	*——*	1.24 (0.47, 3.24)
Rapid descending type with high starting point	2.92 (0.65, 13.15)	0.64 (0.26, 1.57)	0.93 (0.33, 2.58)	1.29 (0.44, 3.75)	2.00 (0.39,10.35)	0.89 (0.39, 2.04)
Stable descending type with medium starting point	1.89 (0.39, 9.19)	1.14 (0.69, 1.90)	1.21 (0.66, 2.21)	1.53 (0.75, 3.09)	2.11 (0.61, 7.28)	1.12 (0.66, 1.90)
***2***						
Stable type at the middle level	1.33 (0.87, 2.03)	1.38** (1.07, 1.78)	1.39** (1.07, 1.81)	1.22 (0.87, 1.69)	1.20 (0.83, 1.74)	1.48*** (1.13, 1.93)
Stable type with low starting point	0.62 (0.16, 2.41)	3.56** (1.02,12.45)	1.51 (0.40, 5.74)	2.42 (0.68, 8.57)	*——*	1.63 (0.63, 4.21)
Rapid descending type with high starting point	2.30 (0.51, 10.42)	1.13 (0.49, 2.63)	1.07 (0.40, 2.91)	1.53 (0.55, 4.29)	1.98 (0.40, 9.73)	1.22 (0.54, 2.74)
Stable descending type with medium starting point	1.58 (0.34, 7.39)	1.40 (0.86, 2.29)	0.96 (0.52, 1.78)	1.95** (1.01, 3.76)	1.95 (0.59, 6.47)	1.35 (0.81, 2.24)
***3***						
Stable type at the middle level	1.89*** (1.25, 2.88)	1.85*** (1.41, 2.44)	1.77*** (1.36, 2.31)	1.63*** (1.16, 2.29)	1.66*** (1.14, 2.41)	2.03*** (1.54, 2.68)
Stable type with low starting point	0.28 (0.05, 1.63)	2.30 (0.60, 8.90)	1.53 (0.38, 6.20)	0.63 (0.14, 2.93)	*——*	0.82 (0.27, 2.48)
Rapid descending type with high starting point	3.71* (0.82, 16.83)	1.36 (0.54, 3.41)	1.56 (0.59, 4.16)	2.07 (0.69, 6.21)	3.72 (0.74,18.64)	1.51 (0.67, 3.42)
Stable descending type with medium starting point	2.93 (0.68, 12.66)	2.31*** (1.39, 3.83)	1.49 (0.81, 2.73)	3.09*** (1.60, 5.98)	1.86 (0.50, 6.94)	2.53*** (1.52, 4.20)
**≥4**						
Stable type at the middle level	2.27*** (1.52, 3.40)	2.08*** (1.56, 2.77)	1.91*** (1.47, 2.48)	1.96*** (1.41, 2.73)	1.57** (1.05, 2.34)	2.55*** (1.96, 3.32)
Stable type with low starting point	1.47 (0.45, 4.79)	5.47*** (1.61,18.52)	4.29** (1.29,14.28)	2.38 (0.69, 8.19)	*——*	2.35* (0.94, 5.90)
Rapid descending type with high starting point	2.52 (0.59, 10.84)	1.27 (0.52, 3.09)	1.36 (0.53, 3.49)	1.37 (0.48, 3.91)	2.48 (0.53,11.56)	1.28 (0.58, 2.82)
Stable descending type with medium starting point	4.05* (0.99, 16.57)	3.24*** (1.98, 5.29)	2.31*** (1.31, 4.08)	3.72*** (1.98, 7.02)	5.58*** (1.69,18.35)	3.17*** (1.95, 5.16)

Note: All control variables are controlled. RRR Relative Risk Ratio.

*, **, *** statistically significant at the 10%, 5% and 1% levels, respectively.

## 4 Discussion

In this study, the average age of respondents was 57.7 years, and the average score for healthy aging was 66.6 in the base period and 65.2 among the four periods. A study analysed healthy aging scores in eight countries with mean age ranging from 61.5 to 77.5 years and healthy aging scores ranging from 56.8 to 76.9, with a combined country score of 67.5 and an overall mean age of 62.9 years (Wu et al., 2020). Compared with the aforementioned results, the average age of the sample was lower, but the same was relatively true for the healthy aging score. This indicates that the current situation regarding healthy aging in China is grim and not optimistic.

Moreover, five types of healthy aging trajectories with different initial levels of health aging scores and slopes were found, which represented five distinct groups of middle-aged and older populations. The results reveals that some healthy aging trajectories maintained a relatively high and stable level, which implied a lower probability of developing chronic diseases and a higher probability of engaging in positive health behaviours or adopting a healthy lifestyle. At the same time, the trajectory of rapid descending type with high starting point showed irreversible downward trends with increasing age, which also represented the target population or priority group for policy interventions, proving that the loss of health could be controlled and intervened or at least delayed.

We found that ACEs were closely associated with the trajectories of healthy aging among older adults in China, which further indicates that it is necessary to examine the issue of healthy aging from the perspective of the life cycle. Further analysis indicated that those suffering from an increased scores of ACEs were more likely to have an unhealthy aging trajectory. It is intriguing that the ACE scores was significantly negatively related to all development trajectories of healthy aging; however, the healthy aging trajectory of the rapid descending type with a high starting point was not affected by which group of ACEs it was in. Nevertheless, ACEs may not be associated with the rapidly descending trend of healthy aging, which may be more associated with the current situation. A possible explanation is that only when the scores of ACEs reaches a certain degree can it affect the development of healthy aging. Additionally, the influence of ACEs on different healthy aging trajectories is regulated by other factors, resulting in different critical values. Compared with the impact of the trajectory slope, ACEs was more likely to affect the level of healthy aging in the base period (starting point or initial stage of healthy aging trajectories). The trajectory showed such a rapid decline, probably caused by major events that occurred at the moment, including sudden serious diseases, accidental injuries, and other factors.

The analysis of the relationship between each ACE and the trajectories of healthy aging revealed that household substance abuse, emotional neglect, parental separation or divorce, and parental death did not have a significant impact on the trajectories of healthy aging. Possible explanations for these findings were as follows. Firstly, individuals may have their emotional needs fulfilled by other family members to some extent due to early occurrence of parental divorce or death. On the flip side, Chinese individuals, adhering to traditional norms, may exhibit limited proficiency in the expression of emotions. Consequently, even when confronted with emotional neglect, it may not necessarily engender adverse consequences. Additionally, to some extent, smoking and alcohol consumption were considered part of social behaviours in China and therefore may not have significantly negative psychological consequences.

Heterogeneity analysis of the relationship between ACEs and the trajectories of healthy aging revealed that, in terms of gender grouping, women's trajectories of healthy aging appeared to be more susceptible to the influence of ACEs compared to men. This may be explained by the inherent sensitivity and vulnerability of women's psychological makeup, often resulting in weaker psychological resilience compared to men (Almuneef M et al., 2016; Evans et al., 2020). Regarding age grouping, ACEs had significant impacts on the middle-aged population characterized by the “stable type at the middle level” and the older population characterized by the “stable descending type with medium starting point”. This reflected that ACEs not only affected the initial level of individuals' healthy aging but also influenced the slopes of healthy aging trajectories. In terms of rural-urban grouping, the trajectory of healthy aging among rural residents shows greater sensitivity to ACEs (Radcliff E et al., 2018; Zhang R et al., 2022). This may be explained by the distinct experiences of rural residents compared to their urban counterparts. Compared to urban residents, in rural areas, there is often limited access to care coordination, social support services, and healthcare opportunities. Consequently, rural families may possess diminished capacity to mitigate and manage the effects of ACEs.

The potential mechanisms linking ACEs and healthy aging trajectories may include biological mechanisms, psychosocial mechanisms, health behaviour mechanisms, and social environmental mechanisms. ACEs may have long-term effects on individuals' biological development, including stress response systems, inflammation increase, neurodevelopment, and gene expression (Slopen N et al., 2016; Berens AE et al., 2017), thereby influencing the trajectories of healthy aging. They may also impact individuals' psychosocial development through negative psychological reactions such as anxiety, depression, and post-traumatic stress disorder (Herzog & Schmahl, 2018; Bourassa KJ et al., 2022), as well as influencing the development of social support networks (Karatekin & Ahluwalia, 2020), coping strategies, and cognitive abilities (Ward K et al., 2022), thereby affecting the trajectory of healthy aging. ACEs could contribute to the formation of unhealthy behavioural patterns, leading to long-term negative effects on individuals' health behaviours and subsequently influencing the trajectory of healthy aging. Additionally, they may directly or indirectly impact individuals' social environments, such as family stability (Liming KW et al., 2021), economic status (Mersky JP et al., 2021), and educational opportunities (Houtepen LC et al., 2020), which can further influence the trajectory of healthy aging.

In conclusion, China has an aging society. We should not only pay attention to the current healthy aging situation of the older population but also promote the realisation of healthy aging from the perspective of the whole population and life cycle. One important measure is avoiding the occurrence of ACE. A study showed that 80.9% of middle-aged and older population in China live in at least one area and 18.0% live in at least four ACEs (Lin et al., 2021). This further proves that ACEs in China are very serious and not optimistic, which deserves our attention. As far as policy implications are concerned, based on the fact that ACEs will have a long-term and lasting impact on healthy aging and its trajectories, to realise healthy aging and deal with the increasingly serious aging problem, we should comprehensively and systematically intervene in all factors affecting health from the perspective of the whole life process, starting from early life and breaking the accumulation process of health disadvantages caused by the actions. This suggests the need to prevent the occurrence of ACEs, reduce potential associated risks of adverse health outcomes, and make active policy interventions after an individual encounters ACEs, as well as implement life-course public healthy aging strategy to promote the trajectories of healthy aging for the better.

## 5 Strengths and limitations

To the best of our knowledge, this is the first study to show a significant negative association between ACEs and trajectories of healthy aging among middle-aged and older individuals in China. Previous studies have found that the trajectories of healthy aging were related to the common environmental aetiology, such as standard of living, urban-rural disparities in residence (Li et al., 2022; Moayyeri et al., 2016; Stephens et al., 2019). Some scholars focused on the relationship between healthy aging trajectories and multimorbidity, education level, household wealth(de la Fuente et al., 2018; McLaughlin et al., 2020; Nguyen et al., 2021), while others studied the association between healthy aging trajectories and health-related lifestyle, such as sleep quality and duration, eating habits, physical activity, smoking and drinking(Daskalopoulou et al., 2019; Gkotzamanis et al., 2022, 2023; Moreno-Agostino et al., 2020; Tampubolon, 2016). This study expands the conclusions of the existing literature. The findings of this study not only provide an important empirical basis for China to implement healthy aging action interventions to promote healthy aging but also expand the research on the trajectories of healthy aging from a new research perspective of the whole population and full life cycle.

This study had some limitations. First, Despite the risk of recall bias in collecting data on the indicators of ACES, previous research has reported the reliability of retrospective measures (Lin et al., 2021). Second, owing to data unavailability, although a weighted ACEs scoring method may enhance measurement precision, we used the cumulative ACEs score (Davis et al., 2014; Slopen et al., 2013). Although the weight consideration was included in this study, more than 60% samples were excluded in this study, which is one of the limitations of this study. This may lead to doubts about the representativeness of the samples and the reliability of the results. Many existing studies have also removed more than 60% samples, which proves the conclusion of this study to some extent. Thirdly, this study also could not completely match the existing Healthy Aging Scale (Wu et al., 2020) and chose to partially replace the indicators of the Healthy Aging Scale based on existing data. The appropriateness of the selection of indicators in this study was proven through reliability and validity tests. Furthermore, this study did not explore the mechanism of ACEs on the developmental trajectories of healthy aging, including mediating effects (mediation analysis). Moreover, this study only demonstrated the correlation between ACEs and healthy aging trajectories. The next step is to further verify the causal relationship between ACEs and healthy aging trajectories.

## 6 Conclusions

This study showed a negative association between ACEs and healthy aging trajectories. We found that scores of ACEs was significantly related to unhealthy aging trajectories, although ACEs did not seem to be associated with a rapidly descending trend when they were treated as categorical variables. The government should promote the realisation of healthy aging from the perspective of the entire population and life cycle, intervene early in life, avoid the occurrence of ACEs as much as possible, or minimize the harm of ACEs as much as possible.

## Ethics approval

The Biomedical Ethics Review Committee of Peking University approved CHARLS, and all participants were required to provide written informed consent. The ethical approval number was IRB00001052-11015.

## Funding

Not applicable.

## Author contributions

JS contributed to the conception and design of the study. JRW completed data analysis and wrote the first draft of the manuscript. HML contributed to supervising data analysis and developing the manuscript. All authors contributed to revising the article and approved the final draft as submitted.

## Ethics approval

The Biomedical Ethics Review Committee of Peking University approved CHARLS, and all participants were required to provide written informed consent. The ethical approval number was IRB00001052-11015.

## Author statement

Ju Sun conceptualised and designed the study. Jingru Wang acquired and analysed the data. Jingru Wang wrote the first draft of the article under the supervision of Ju Sun and Haomiao Li. Haomiao Li critically revised the draft. All authors reviewed and approved the final version of the article.

## Data Availability

Data will be made available on request.
