# Supplementary material for: Are adverse childhood experiences associated with trajectories of healthy aging? Evidence from China
Source: SSM Popul Health. 2023 Aug 26;24:101501. doi: 10.1016/j.ssmph.2023.101501 (PMC10492199; doi:10.1016/j.ssmph.2023.101501)
Supplement: Multimedia component 1 [file mmc1.docx]

**Are adverse childhood experiences associated with trajectories of healthy aging? Evidence from China**

**Supplementary figure**

Figure S1 Flow chart of sample selection

Figure S2 Using Directed Acyclic Graphs (DAGs) to assess variables

Table S1 Items to calculate healthy aging

Table S2 Questionnaire Items of Each Adverse Childhood Experience Indicator

Table S3 Group characteristics corresponding to five kinds of healthy aging trajectories

Table S4 The association between each ACEs items and trajectories of healthy aging based on multinomial logistic regression on the basis of sampling weight (Reference: stable type with high starting point)

Table S5 sensitivity analyses

Figure S1 Flow chart of sample selection


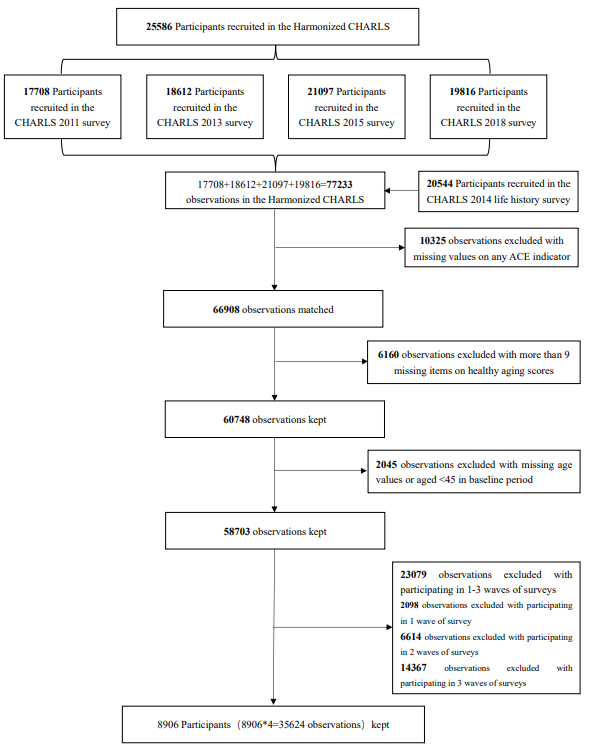


Figure S2 Using Directed Acyclic Graphs (DAGs) to assess variables


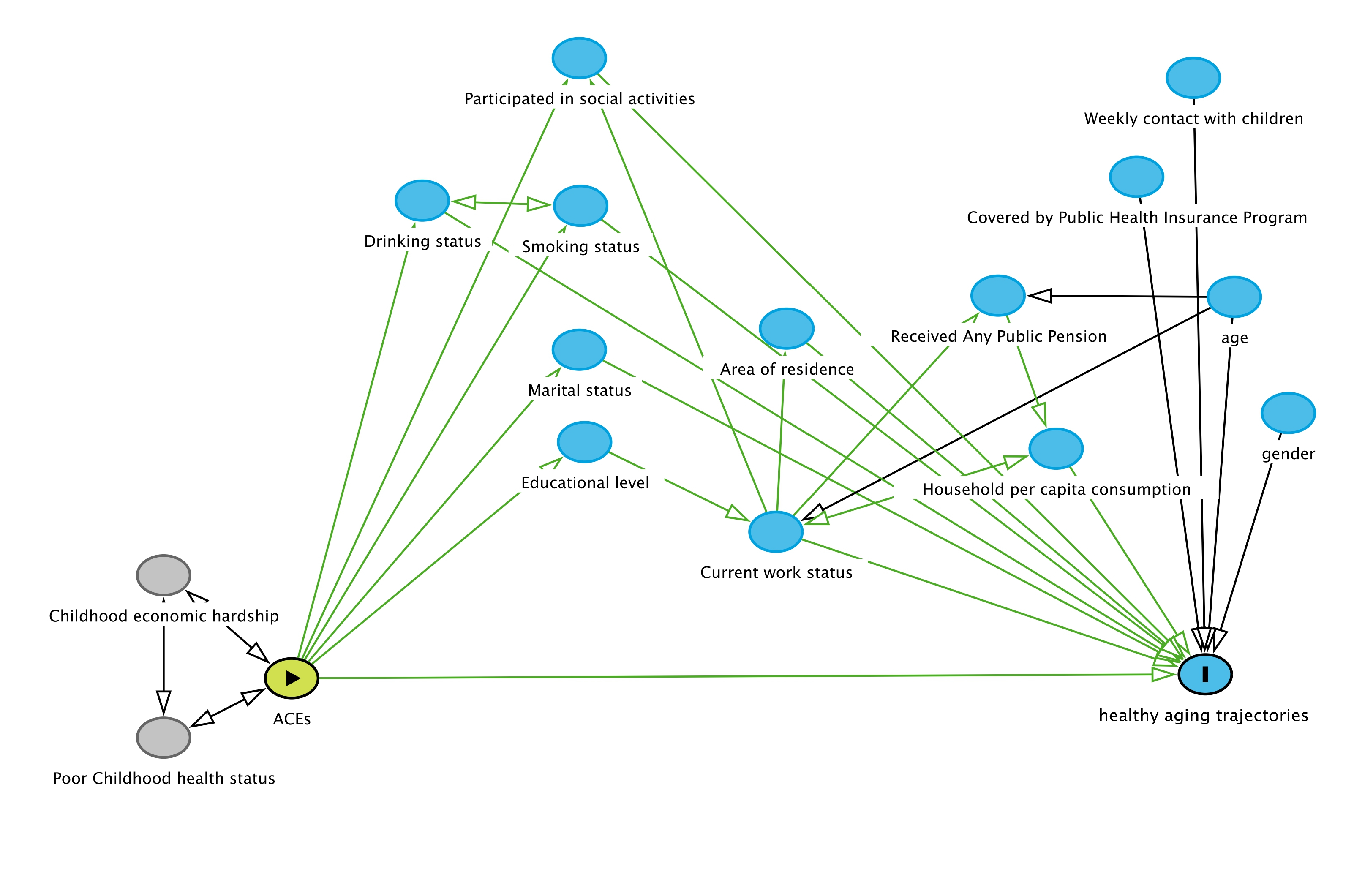


Table S1 Items to calculate healthy aging

| Domains | Presence or absence of difficulties |
| --- | --- |
| Cognition | Memory |
|  | Orientation in time |
|  | Draw assign picture |
|  | Immediate recall |
|  | Delayed recall |
|  | Serial 7's test |
| Psychology symptoms（CESD-10） | Bothered by little things |
|  | Felt depressed |
|  | Had trouble keeping my mind on what I was doing |
|  | Felt fearful |
|  | Sleep was restless |
|  | Felt lonely |
|  | Felt that everything I did was an effort |
|  | Was happy |
|  | Felt hopeful about the future |
| Vitality | Experiences some degree of pain |
|  | Having high level of energy |
|  | Controlling Urination and Defecation |
| Sensory functions | Eyesight using glasses or lens as usual |
|  | Near vision |
|  | Far vision |
|  | Hearing in general |
| Mobility | Stooping, kneeling or crouching |
|  | Lifting or carrying weights |
|  | Climbing stairs |
|  | Getting up from sitting down |
|  | Reaching or extending arms |
|  | Running or jogging about 1 km |
|  | Picking up things with fingers |
| ADL | Getting in or out of bed |
|  | Bathing or showering |
|  | Getting dressed |
|  | Walking 100 meters |
|  | Using the toilet |
|  | Eating |
| IADL | Doing housework |
|  | Shopping for groceries |
|  | Walking 1 km |
|  | preparing meals |
|  | Managing money, bills or expenses |
|  | Taking medications |

Note: 0 = Presence of difficulties, 1 = Absence of difficulties.

Table S2 Questionnaire Items of Each Adverse Childhood Experience Indicator

| ACEs Domain | Questionnaire Items |
| --- | --- |
| Physical abuse | When you were growing up, did your female/male guardian ever hit you? (often^a^, sometimes^a^, rarely, or never) |
| Emotional neglect | How much love and affection did your female guardian give you while you were growing up? (often, sometimes, rarely^a^, or never^a^) |
|  | How much effort did your female guardian put into watching over you? (a lot, some, a little^a^, or not at all^a^) |
| Household substance abuse | During the years you were growing up, did your female/male guardian ever have alcoholism or drug? (yes^a^ or no) |
| Household mental illness | Did your female/male guardian have abnormality of mind when you were young? (yes^a^ or no) |
|  | During the years you were growing up, had your female/male guardian often showed continued signs of sadness or depression? (during all^a^, most^a^, some, or only a little of the childhood) |
| Domestic violence | Have your father/mother ever beat up your mother/father? (often^a^, sometimes^a^, not very often, or never) |
| Incarcerated household member | During the years you were growing up, have your female/male guardian ever been arrested or sent to prison? (yes^a^ or no)  1. Have trouble holding a job  2. Often not tell a truth  3. Often get into physical fights  4. Involved in criminal activities like burglary or selling stolen property  5. Arrested or sent to prison  6. None of that |
| Parental separation or divorce | Were your biological parents divorced (including long separation due to emotional problems) before you were 17 years? (yes^a^ or no) |
| Unsafe neighborhood | Was it safe being out alone at night in the neighborhood where you lived as a child? (very safe, somewhat safe, not very safe^a^, or not safe at all^a^) |
| Bullying | When you were a child, how often were you picked on or bullied by kids in your neighborhood? (often^a^, sometimes^a^, not very often, or never) |
|  | When you were a child, how often were you picked on or bullied by kids in your school? (often^a^, sometimes^a^, not very often, or never) |
| Parental death^b^ | Either of the parents was dead before participant was 17 years. (yes^a^ or no) |
| Sibling death^c^ | Any of the siblings was dead before participant was 17 years. (yes^a^ or no) |
| Parental disability | Did your female/male guardian have a long time being sick on bed when you were young? (yes^a^ or no) |
|  | Did your female/male guardian have a serious deformity when you were young? (yes^a^ or no) |

Abbreviation: ACEs: adverse childhood experiences.

^a^ Answers indicate thresholds for ACEs.

^b^ Calculated based on dates of birth and their parental death.

^c^ Calculated based on dates of birth and their sibling's death.

Table S3 Group characteristics corresponding to five kinds of healthy aging trajectories

| **Variables** | Mean±SD/ N(%) | | | | |
| --- | --- | --- | --- | --- | --- |
|  | stable type with high starting point | Stable type at the middle level | Stable type with low  starting point | Rapid descending type with high starting point | Stable descending type with medium starting point |
| **ACE scores** | 2.57±1.78 | 2.96±1.93 | 3.61±2.51 | 2.88±1.93 | 3.28±2.05 |
| **ACEs group** | | | | | |
| 0 | 439  (10.00%) | 298  (7.97%) | 6  (4.84%) | 11  (7.59%) | 33  (6.55%) |
| 1 | 930  (21.17%) | 604  (16.15%) | 21  (16.94%) | 26  (17.93%) | 75  (14.88%) |
| 2 | 1012  (23.04%) | 791  (21.14%) | 28  (22.58%) | 33  (22.76%) | 89  (17.66%) |
| 3 | 802  (18.26%) | 762  (20.37%) | 12  (9.68%) | 29  (20.00%) | 96  (19.05%) |
| ≥4 | 1209  (27.53%) | 1286  (34.38%) | 57  (45.97%) | 46  (31.72%) | 211  (41.87%) |
| **Age** | 57.82± 8.20 | 57.27± 8.07 | 58.14± 8.38 | 59.88± 8.87 | 59.29± 8.1 |
| **Gender** | | | | | |
| male | 3029  (68.97%) | 929  (24.83%) | 47  (37.90%) | 70  (48.28%) | 68  (13.49%) |
| female | 1363  (31.03%) | 2812  (75.17%) | 77  (62.10%) | 75  (51.72%) | 436  (86.51%) |
| **Marital status** | | | | | |
| Married with spouse present | 3915  (89.14%) | 3129  (83.64%) | 101  (81.45%) | 120  (82.76%) | 393  (77.98%) |
| others | 477  (10.86%) | 612  (16.36%) | 23  (18.55%) | 25  (17.24%) | 111  (22.02%) |
| **Educational level** | | | | | |
| Less than lower secondary education | 3694  (84.11%) | 3523  (94.17%) | 121  (97.58%) | 139  (95.86%) | 495  (98.21%) |
| Upper secondary & vocational training | 614  (13.98%) | 207  (5.53%) | 3  (2.42%) | 6  (4.14%) | 7  (1.39%) |
| Tertiary education | 84  (1.91%) | 11  (0.29%) | —— | —— | 2  (0.40%) |
| **Area of residence** | | | | | |
| Rural | 2617  (59.59%) | 2622  (70.09%) | 91  (73.39%) | 109  (75.17%) | 407  (80.75%) |
| Urban | 1775  (40.41%) | 1119  (29.91%) | 33  (26.61%) | 36  (24.83%) | 97  (19.25%) |
| **Current work status** | | | | | |
| Not working | 1168  (26.59%) | 1241  (33.17%) | 74  (59.68%) | 52  (35.86%) | 200  (39.68%) |
| Working | 3224  (73.41%) | 2500  (66.83%) | 50  (40.32%) | 93  (64.14%) | 304  (60.32%) |
| **Childhood economic hardship** | | | | | |
| Yes | 1552  (35.34%) | 1606  (42.93%) | 75  (60.48%) | 68  (46.90%) | 288  (57.14%) |
| No | 2840  (64.66%) | 2135  (57.07%) | 49  (39.52%) | 77  (53.10%) | 216  (42.86%) |
| **Poor Childhood health status** | | | | | |
| Yes | 387  (8.81%) | 572  (15.29%) | 31  (25.00%) | 20  (13.79%) | 125  (24.80%) |
| No | 4005  (91.19%) | 3169  (84.71%) | 93  (75.00%) | 125  (86.21%) | 379  (75.20%) |
| **Poor Self-rated Health** | | | | | |
| Yes | 485  (11.04%) | 1421  (37.98%) | 105  (84.68%) | 29  (20.00%) | 315  (62.50%) |
| No | 3907  (88.96%) | 2320  (62.02%) | 19  (15.32%) | 116  (80.00%) | 189  (37.50%) |
| **Chronic disease** | | | | | |
| None | 1869  (42.55%) | 868  (23.20%) | 12  (9.68%) | 37  (25.52%) | 57  (11.31%) |
| Yes | 1381  (31.44%) | 1104  (29.51%) | 22  (17.74%) | 45  (31.03%) | 124  (24.60%) |
| Multimorbidity (Morbidity) | 1142  (26.00%) | 1769  (47.29%) | 90  (72.58%) | 63  (43.45%) | 323  (64.09%) |
| **Covered by Public Health Insurance Program** | | | | | |
| None | 273  (6.22%) | 221  (5.91%) | 4  (3.23%) | 12  (8.28%) | 35  (6.94%) |
| Yes | 4119  (93.78%) | 3520  (94.09%) | 120  (96.77%) | 133  (91.72%) | 469  (93.06%) |
| **Received Any Public Pension** | | | | | |
| None | 3573  (81.35%) | 3148  (84.15%) | 108  (87.10%) | 129  (88.97%) | 420  (83.33%) |
| yes | 819  (18.65%) | 593  (15.85%) | 16  (12.90%) | 16  (11.03%) | 84  (16.67%) |
| **Take Household per capita consumption´s log** | 7.36±3.07 | 7.21±3.02 | 6.89±3.31 | 7.31±2.80 | 7.180±2.99 |
| **Smoking status** | | | | | |
| Never | 2065  (47.02%) | 2824  (75.49%) | 79  (63.71%) | 90  (62.07%) | 411  (81.55%) |
| Quit now | 459  (10.45%) | 219  (5.85%) | 14  (11.29%) | 13  (8.97%) | 22  (4.37%) |
| Still | 1868  (42.53%) | 698  (18.66%) | 31  (25.00%) | 42  (28.97%) | 71  (14.09%) |
| **Drinking status** | | | | | |
| Never | 2081  (47.38%) | 2619  (70.01%) | 84  (67.74%) | 85  (58.62%) | 374  (74.21%) |
| Quit now | 462  (10.52%) | 367  (9.81%) | 16  (12.90%) | 13  (8.97%) | 55  (10.91%) |
| Still | 1849  (42.10%) | 755  (20.18%) | 24  (19.35%) | 47  (32.41%) | 75  (14.88%) |
| **Participated in social activities** | | | | | |
| None | 2169  (49.39%) | 2102  (56.19%) | 92  (74.19%) | 84  (57.93%) | 305  (60.52%) |
| yes | 2223  (50.61%) | 1639  (43.81%) | 32  (25.81%) | 61  (42.07%) | 199  (39.48%) |
| **Weekly contact with children** | | | | | |
| None | 369  (8.40%) | 367  (9.81%) | 23  (18.55%) | 20  (13.79%) | 54  (10.71%) |
| yes | 4023  (91.60%) | 3374  (90.19%) | 101  (81.45%) | 125  (86.21%) | 450  (89.29%) |
| N | 4392 | 3741 | 124 | 145 | 504 |

Table S4 The association between each ACEs items and trajectory of healthy aging based on multinomial logistic regression on the basis of sampling weight (Reference: stable type with high starting point)

| **ACEs items** | Stable type at the middle level | Stable type with low  starting point | Rapid descending type with high starting point | Stable descending type with medium starting point |
| --- | --- | --- | --- | --- |
|  | RRR  (95% CI) | RRR  (95% CI) | RRR  (95% CI) | RRR  (95% CI) |
| Physical abuse | 1.39***  (1.21,1.60) | 1.65**  (1.08,2.54) | 1.34  (0.90,2.02) | 1.62***  (1.26,2.08) |
| Emotional neglect | 1.10  (0.97,1.24) | 0.93  (0.61,1.41) | 1.02  (0.69,1.50) | 1.14  (0.91,1.43) |
| Household substance abuse | 1.11*  (0.99,1.25) | 1.21  (0.81,1.80) | 0.83  (0.58,1.21) | 0.90  (0.73,1.12) |
| Household mental illness | 2.00***  (1.74,2.31) | 3.80***  (2.49,5.80) | 1.70**  (1.02,2.85) | 3.60***  (2.83,4.57) |
| Domestic violence | 1.35***  (1.15,1.59) | 1.67**  (1.06,2.64) | 0.60*  (0.36,1.02) | 1.39**  (1.05,1.84) |
| Incarcerated household member | 0.49  (0.19,1.30) | 2.62e-11***  (1.26e-11,5.45e-11) | 1.69e-11***  (8.41e-12, 3.40e-11) | 1.91  (0.50,7.25) |
| Parental separation or divorce | 1.57  (0.86,2.87) | 2.28  (0.26,19.60) | 3.67  (0.78,17.38) | 3.51**  (1.34,9.23) |
| Unsafe neighborhood | 1.80***  (1.47,2.20) | 2.32***  (1.32,4.10) | 1.31  (0.71,2.42) | 1.99***  (1.40,2.81) |
| Bullying | 1.55***  (1.35,1.77) | 1.48*  (0.97,2.26) | 1.23  (0.81,1.87) | 1.48***  (1.16,1.90) |
| Parental death | 1.10  (0.95,1.27) | 0.98  (0.60,1.62) | 1.22  (0.77,1.93) | 0.98  (0.74,1.30) |
| Sibling death | 1.23***  (1.06,1.43) | 1.17  (0.75,1.83) | 1.14  (0.73,1.79) | 1.47***  (1.14,1.90) |
| Parental disability | 1.51***  (1.31,1.74) | 3.14***  (2.09,4.73) | 1.76***  (1.16,2.66) | 2.54***  (2.00,3.22) |
| **Covariates** | controlled | controlled | controlled | controlled |

*Note: RRR Relative Risk Ratio.* **, **, *** statistically significant at the 10%, 5% and 1% levels, respectively.*

Table S5 sensitivity analyses (Reference: stable type with high starting point)

|  | **The association between ACEs and trajectory of healthy aging with control variables including self-rated health and chronic diseases using 2011 data** | | | | | | | | | | **The association between ACEs and trajectory of healthy aging with control variables including self-rated health and chronic diseases using 2018 data** | | | | | | | | |
| --- | --- | --- | --- | --- | --- | --- | --- | --- | --- | --- | --- | --- | --- | --- | --- | --- | --- | --- | --- |
| **Variable** | Stable type at the middle level | | Stable type with low starting point | | Rapid descending type with high starting point | | Stable descending type with medium starting point | | Stable type at the middle level | | | Stable type with low starting point | | Rapid descending type with high starting point | | Stable descending type with medium starting point | | Random effects model | |
|  | RRR (95% CI) | RRR (95% CI) | RRR (95% CI) | RRR (95% CI) | RRR (95% CI) | RRR (95% CI) | RRR (95% CI) | RRR (95% CI) | RRR (95% CI) | RRR (95% CI) | | RRR (95% CI) | RRR (95% CI) | RRR (95% CI) | RRR (95% CI) | RRR (95% CI) | RRR (95% CI) | RRR(95% CI) | RRR (95% CI) |
| **ACE scores** | -- | 1.16^***^  (1.12,1.20) | -- | 1.29^***^  (1.15,1.44) | -- | 1.07  (0.98,1.18) | -- | 1.25^***^  (1.18,1.33) | -- | 1.17^***^  (1.14,1.21) | | -- | 1.30^***^  (1.19,1.43) | -- | 1.11^**^  (1.01,1.21) | -- | 1.25^***^  (1.19,1.33) | -- | -0.79^***^  (0.05) |
| **Aces（Categorical Variable, Ref: aces=0）** | | | | | | | | | | | | | | | | | | | |
| **1** | 0.96  (0.76,1.22) | -- | 1.48  (0.56,3.86) | -- | 1.07  (0.51,2.25) | -- | 1.13  (0.69,1.84) | -- | 0.96  (0.77, 1.19) | -- | | 1.55  (0.61,3.97) | -- | 1.11  (0.54, 2.32) | -- | 1.16  (0.72, 1.89) | -- | -0.30  (0.38) | -- |
| **2** | 1.21^*^  (0.96,1.52) | -- | 1.59  (0.62,4.07) | -- | 1.24  (0.60,2.54) | -- | 1.14  (0.71,1.83) | -- | 1.28**  (1.04, 1.58) | -- | | 1.97  (0.79, 4.91) | -- | 1.37  (0.67, 2.80) | -- | 1.34  (0.83, 2.15) | -- | -1.41***  (0.37) | -- |
| **3** | 1.64^***^  (1.30,2.08) | -- | 0.85  (0.29,2.49) | -- | 1.80  (0.86,3.77) | -- | 1.85^**^  (1.14,3.00) | -- | 1.69***  (1.36, 2.10) | -- | | 1.09  (0.39, 3.01) | -- | 1.58  (0.76,3.27) | -- | 1.94***  (1.20, 3.12) | -- | -2.22***  (0.38) | -- |
| **≥4** | 1.82^***^  (1.44,2.30) | -- | 2.64^**^  (1.06,6.53) | -- | 1.33  (0.66,2.68) | -- | 2.53^***^  (1.60,4.00) | -- | 1.97***  (1.60, 2.42) | -- | | 3.19**  (1.31, 7.75) | -- | 1.62  (0.80, 3.26) | -- | 2.87***  (1.83,4.49) | -- | -3.65***  (0.36) | -- |

*Note: RRR Relative Risk Ratio.*

**, **, *** statistically significant at the 10%, 5% and 1% levels, respectively.*

*All control variables are controlled.*
